# Elebsiran and pegylated-IFNα: Progress toward a functional cure for chronic hepatitis B

**DOI:** 10.1016/j.jhepr.2026.101741

**Published:** 2026-02-26

**Authors:** Sara Battistella, Ulrike Protzer, Xavier Forns

**Affiliations:** 1 Liver Unit, Hospital Clinic, Universitat de Barcelona, IDIBAPS, Barcelona, Spain; 2 Centro de Investigación Biomédica en Red de Enfermedades Hepáticas y Digestivas (CIBEREHD), Spain; 3 Gastroenterology Unit, Department of Surgery, Oncology and Gastroenterology, University of Padua, Italy; 4 Institute of Virology, Technical University of Munich, School of Medicine and Health/Helmholtz Munich, Germany; 5 German Center for Infection Research (DZIF), Munich Partner Site, Germany

Wong, G.LH., Yuen, MF., Lin, B. *et al.* Elebsiran and PEG-IFNα for chronic hepatitis B infection: a partially randomized, open-label, phase 2 trial. *Nat Med* (2025). https://doi.org/10.1038/s41591-025-04049-z

Functional cure, defined as sustained HBsAg loss 24 weeks after the end of treatment (EOT), remains the ultimate goal for patients with chronic hepatitis B (CHB).^1^ Achieving this endpoint requires: (1) suppression of viral replication, (2) cessation of viral protein production, and (3) restoration of HBV-specific immune responses. Combination regimens will likely be necessary to achieve both viral suppression and immune control.^1^

More than 30 agents are currently under development. Among these, small interfering RNAs (siRNAs) have emerged as potent inhibitors of viral antigen production; however, they rarely result in HBsAg loss. New CRISPR-based gene or cccDNA epigenetic silencing approaches have just entered clinical trials but face challenges related to efficient targeting, HBV-DNA integration, off-target effects, and hepatotoxicity.

Elebsiran, an siRNA targeting a highly conserved region of the HBx gene, has demonstrated meaningful HBsAg reductions as monotherapy but showed greater HBsAg declines and seroclearance rates when combined with PEG-IFNα.^2^

In the phase II ENSURE trial, published in Nature Medicine by *Wong et al.*,^3^ the combination of elebsiran+PEG-IFNα *vs.* PEG-IFNα alone was evaluated in 55 patients with baseline HBsAg of 100-3,000 IU/ml and undetectable HBV-DNA under nucleos(t)ide analogue therapy. While only 5.6% of participants receiving PEG-IFNα monotherapy achieved HBsAg loss at EOT and 24 weeks post-EOT, the combination of elebsiran+PEG-IFNα yielded markedly higher rates – 29.7% at EOT and 27% at 24 weeks post-EOT. These data confirm that the antigen suppression by siRNA enhances the therapeutic impact of PEG-IFNα, which represses cccDNA but also has an immune modulatory effect.^4^

Supporting evidence is derived from other combination therapy trials. In the PIRANGA trial,^5^ treatment with siRNA+PEG-IFNα for 48 weeks resulted in HBsAg loss in 23% of patients at 24 weeks post-EOT, whereas in IM-PROVE I, siRNA followed by 24 weeks of PEG-IFNα (with or without continuation of siRNA) resulted in a 24% HBsAg loss rate at the same time point.^6^ Across these studies, prolonged PEG-IFNα exposure and lower baseline HBsAg levels predicted response, emphasizing that durable immune stimulation is critical for treatment efficacy.

The most compelling signal from the ENSURE trial^3^ came from cohort 4, consisting of 31 patients previously exposed to the therapeutic vaccine BRII-179, who received elebsiran+PEG-IFNα. In this cohort, HBsAg loss reached 41.9% at EOT and 29% 24 weeks post-EOT. Remarkably, among individuals who had responded to BRII-179, defined as anti-HBs >10 IU/L, these rates rose to 57.9% and 42.1%, respectively. This suggests that, on one hand, therapeutic vaccination plays a role in priming the immune system, and, on the other hand, can help identify patients capable of immune restoration with subsequent PEG-IFNα therapy. This is important because, despite encouraging efficacy, combination regimens including PEG-IFNα increase the risk of side effects. In the ENSURE trial, 90% of patients experienced PEG-IFNα-related adverse events. Elebsiran-related events occurred in 48.5% of patients, with no serious toxicity.

From a pathogen perspective, siRNA-mediated antigen reduction may restore anti-HBs B-cell function, consistent with observations that combined treatment with BRII-179 and siRNA led to a more sustained anti-HBs response than the vaccine alone.^7^ Indeed, beyond HBsAg clearance, the combination therapy appears to help restore antiviral immunity. In patients receiving BRII-179 + elebsiran ± PEG-IFNα,^7^ a robust expansion of Pre-S-specific CD4^+^ T cells was observed, although HBs-specific CD8^+^ T cells remained largely dysfunctional. Antigen-dependent differences in HBV-specific CD8^+^ T-cell exhaustion may explain, at least in part, these findings. Indeed, CD8^+^ T cells tend to better recognize the less abundant viral core and polymerase antigens.^8^ Nevertheless, the induction of strong and persistent CD4^+^ T-cell responses by BRII-179 may enable future prime-boost strategies, pairing it with agents that preferentially activate CD8^+^ T cells.^9^

Finally, current antiviral therapies still do not eliminate cccDNA or integrated HBV-DNA. Achieving sterilizing cure will likely require a restoration of CD8^+^ T-cell immunity to eliminate cells with integrated HBV-DNA. Future strategies should consider a sequential design, with an initial antigen reduction phase with siRNA, antisense oligonucleotide, or a monoclonal antibody to lower the level of immune tolerance,^10^ followed by immune stimulation using PEG-IFNα, a therapeutic vaccine, or immunotherapies to boost the HBV-specific immune response (Fig. 1).

**Fig. 1:**
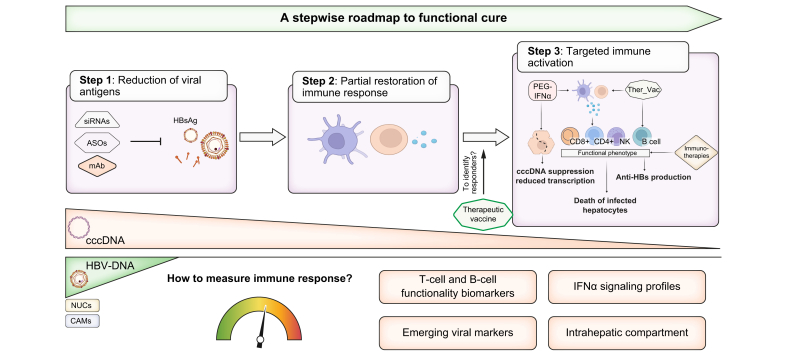
A multistep strategy for functional cure of chronic hepatitis B. ASOs, antisense oligonucleotides; CAMs, capsid assembly modulators; NUCs, nucleos(t)ide analogues; mAb, monoclonal antibody.

However, it will be crucial to “measure” immune responses, not merely through anti-HBs titers, but also by functional immune cell assays. Translational immunology studies are needed to define biomarkers that predict the durability of an HBsAg loss, including phenotype and function of HBV-specific T and B cells, IFNα signaling profile, and emerging viral biomarkers. The fact that liver-specific immune cells are poorly represented in peripheral blood underscores the value of minimally invasive approaches, such as fine-needle aspiration biopsy, for comprehensive profiling of intrahepatic populations.

The ENSURE study demonstrated that viral antigen reduction, coupled with PEG-IFNα, achieves significantly higher rates of functional cure compared to PEG-IFNα alone, particularly in previous responders to therapeutic vaccine. Moving forward, precision immunotherapy, guided by immune biomarkers and patient stratification, should replace empiric combination strategies. Restoring functional HBV-specific immunity, rather than merely suppressing replication, is now the defining frontier in the pursuit of HBV cure.

## Financial support

XF and UP were supported by EU Horizon 2020 Consortium TherVacB

## Authors’ contributions

All authors contributed equally.

## Conflict of interest

The authors of this study declare that they do not have any conflict of interest. Please refer to the accompanying ICMJE disclosure forms for further details.
